# Relative Importance of Current and Past Landscape Structure and Local Habitat Conditions for Plant Species Richness in Dry Grassland-Like Forest Openings

**DOI:** 10.1371/journal.pone.0097110

**Published:** 2014-05-08

**Authors:** Iveta Husáková, Zuzana Münzbergová

**Affiliations:** 1 Department of Botany, Faculty of Science, Charles University, Prague, Czech Republic; 2 Institute of Botany, Academy of Sciences, Průhonice, Czech Republic; University of Tartu, Estonia

## Abstract

In fragmented landscapes, plant species richness may depend not only on local habitat conditions but also on landscape structure. In addition, both present and past landscape structure may be important for species richness. There are, however, only a few studies that have investigated the relative importance of all of these factors. The aim of this study was to examine the effect of current and past landscape structures and habitat conditions on species richness at dry grassland-like forest openings in a forested landscape and to assess their relative importance for species richness. We analyzed information on past and present landscape structures using aerial photographs from 1938, 1973, 1988, 2000 and 2007. We calculated the area of each locality and its isolation in the present and in the past and the continuity of localities in GIS. At each locality, we recorded all vascular plant species (296 species in 110 forest openings) and information on abiotic conditions of the localities. We found that the current species richness of the forest openings was significantly determined by local habitat conditions as well as by landscape structure in the present and in the past. The highest species richness was observed on larger and more heterogeneous localities with rocks and shallow soils, which were already large and well connected to other localities in 1938. The changes in the landscape structure in the past can thus have strong effects on current species richness. Future studies attempting to understand determinants of species diversity in fragmented landscapes should also include data on past landscape structure, as it may in fact be more important than the present structure.

## Introduction

At the landscape level, the distribution and dynamics of plant species are determined not only by local habitat conditions but also by landscape structure and its changes over time [1], [2]. In a landscape, new habitats can appear, and the existing habitats can disappear. In the European landscape, many previously opened habitats that have been maintained by human activity, such as mowing or keeping grazing animals, are slowly disappearing and are consequently becoming smaller and more fragmented [3]–[5].

As a consequence of landscape fragmentation, numerous species populations are becoming small and isolated. Species in small populations are strongly influenced by demographic and environmental stochasticity and are thus prone to extinction [6]–[10]. Moreover, reduced connectivity between the patches can limit the spread of the species due to the increasing distance or presence of barriers between patches and therefore lead to a lower possibility of recolonization of those patches [11], [12]. All of these processes can result in a reduced habitat species richness. To understand the importance of landscape structure changes for species richness, we need to separate these effects from other factors driving the species richness of a habitat.

Among the basic drivers of habitat species richness are the habitat area and isolation. The existence of a relationship between habitat area and species richness was traditionally postulated by the equilibrium theory of island biogeography [13]. A positive species-area relationship has been demonstrated by a large number of studies (e.g., [14]–[17]) and has been explained by several theories (see [13], [18], [19]). Similarly, numerous studies have demonstrated the negative effects of habitat isolation on species richness (e.g., [7], [8], [17], [20], [21]), which also provided a range of explanations for this pattern (see [3], [12], [22], [23]).

The species richness of a habitat patch can also depend on habitat conditions at the sites, such as the type of substrate, type of vegetation, soil depth, slope [23]–[25], insolation or geology [26], soil pH [24], [27], and, in some cases, the disturbance regime [8] or habitat heterogeneity in terms of land use history [26], [28]. However, the species richness of a habitat patch can also depend on the habitat diversity in the surrounding landscape [29], [30]. This last parameter is important especially at a larger spatial scale and in the landscape with more habitat types, which are common for agricultural landscapes with different land use types.

The studies assessing the effects of current habitat size, isolation and habitat conditions on species richness implicitly assume that the system is in equilibrium so the species richness can reflect the status of the landscape [31]. Such an assumption is likely to be valid in oceanic islands that have maintained their size and isolation for thousands or even millions of years, where the habitat conditions at these sites are relatively stable [14], [19], [32]. In the mainland, the landscape is much more dynamic, leading to strong changes in habitat size and isolation over time. Therefore, assuming that the distribution of plant species is in equilibrium may be misleading (e.g., [1], [2], [31], [33], [34]). To understand the drivers of plant species richness in such a landscape, we need to consider the history of the landscape. In this study, we considered the landscape history of only the last one hundred years and did not use a longer-term perspective. Thus, we do not include the processes of speciation [35], [36].

The effect of the last few hundred years of landscape history on plant species richness has been studied mainly in forest fragments [6], [11], [37], [38]. Studies looking at other types of habitats are relatively rare, were performed in rural or agricultural landscapes and focused mainly on changes of land use in particular habitats [26], [28], [39]–[42]. Much less is, however, known about the effects of the past landscape structure (in terms of habitat size and isolation) on patch-scale species richness in fragmented grassland systems [5], [43], [44].

The above-mentioned studies have demonstrated that the plant species richness in fragmented localities is a result of the current and the past landscape structure and their habitat conditions. In spite of the relatively high number of studies addressing some of these factors, there is surprisingly little information on the relative importance of all of these factors, especially in dry grassland communities. Such knowledge is however crucial for effective conservation of plant species richness.

The aim of this study was to assess the relative importance of current and past landscape structures and habitat conditions for plant species richness in dry grassland-like forest openings, below noted only as “forest openings”, in the forested landscape of Křivoklátsko Biosphere Reserve, Czech Republic. In this area, the forest openings are places with the highest plant and animal species richness and are thus of conservation interest [45]. During the last century, these places experienced several changes in the landscape structure, which was at first larger and more connected but is now smaller and more isolated. The main factors responsible for changes in the landscape are shrub invasions likely initiated by increased nutrient deposition since the 1990s [46], [47] and outbreak of hoofed game (Ungulates), especially mouflons (*Ovis musimon*), in the region in the 1970s and 1980s [46], [48]. To identify determinants of patch-scale plant species richness in this landscape, we asked the following questions: (i) Which factors are responsible for plant species richness in forest openings in the forest matrix? (ii) What is the relative importance of three groups of factors, namely, current landscape structure, past landscape structure and habitat conditions, for plant species richness in the patches?

To answer these questions, we mapped all of the current forest openings and collected data on plant species distribution and abiotic conditions at each locality. We analyzed information on past and present landscape structure using aerial photographs from 1938, 1973, 1988, 2000 and 2007. In GIS we calculated the total area of each locality and its isolation in the present as well as its continuity, isolation and area in the past and also some parameters describing habitat conditions. We assessed the relative importance of all of these factors for species richness.

## Methods

Permits and approvals for the field work were obtained from the Protected Landscape Area (PLA) Administration Křivoklátsko. We did not sample any of the protected vascular plant species or perform any experiments with them.

### The Study System

**Figure 1:**
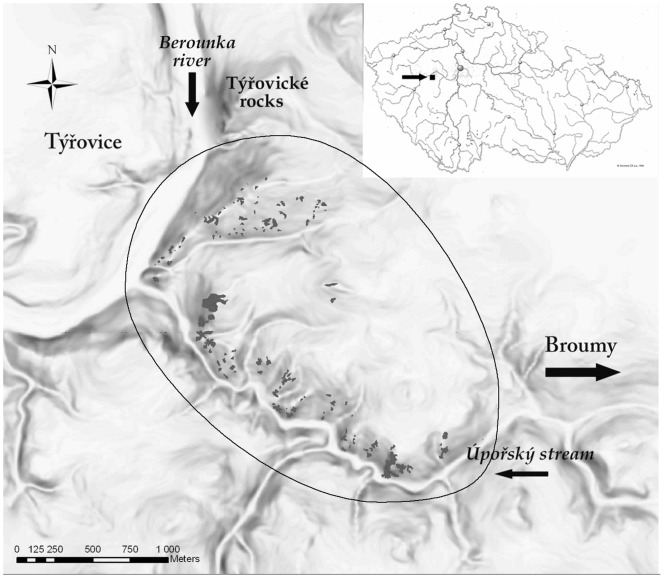
Study area with all studied localities. Study area with all studied localities (gray) in Site of Community Importance (SCI) Týřov (49°58′10″ N, 13°48′40″ E) and the position of study area within the Czech Republic. The line defines the border of our study area.

The study was performed in the Protected Landscape Area and Biosphere Reserve Křivoklátsko in the Czech Republic, specifically at the Site of Community Importance (SCI) Týřov (49°58′10″ N, 13°48′40″ E) (Figure 1). The study area was approximately 4 km^2^ and mainly forested. Approximately 2% of the area was represented by forest openings, a mean area of 635 m^2^ and a mean nearest distance between localities of 48 m. In the study region, we recorded 110 forest openings with the area ranging from 20 m^2^ to 11,123 m^2^. The vegetation of these localities is composed of a specific mosaic of different xerophilous vegetation units ranging from chasmophytic vegetation of rocks and rock crevices (*Asplenietea trichomanis, Festuco-Brometea*), vegetation of primitive soils (*Sedo-Scleranthetea*) and mobile screes (*Thlaspietea rotundifolii*) to vegetation of dry grasslands (*Festuco-Brometea*) and dry herbaceous fringes (*Trifolio-Geranietea*) [49]. At some localities, the vegetation includes ruderal plant communities (*Artemisietea vulgaris, Chenopodietea*) [49]. The surrounding forests are represented by ancient dry acidophilous oak forests (*Quercion petraeae*, *Genisto germanicae-Quercion*), which have been barely managed due to their specific and badly accessible position [48]. Additionally, all of our localities were not managed (no removal of young trees and shrubs colonizing grassland areas, no mowing, grazing cattle, etc.).

**Figure 2:**
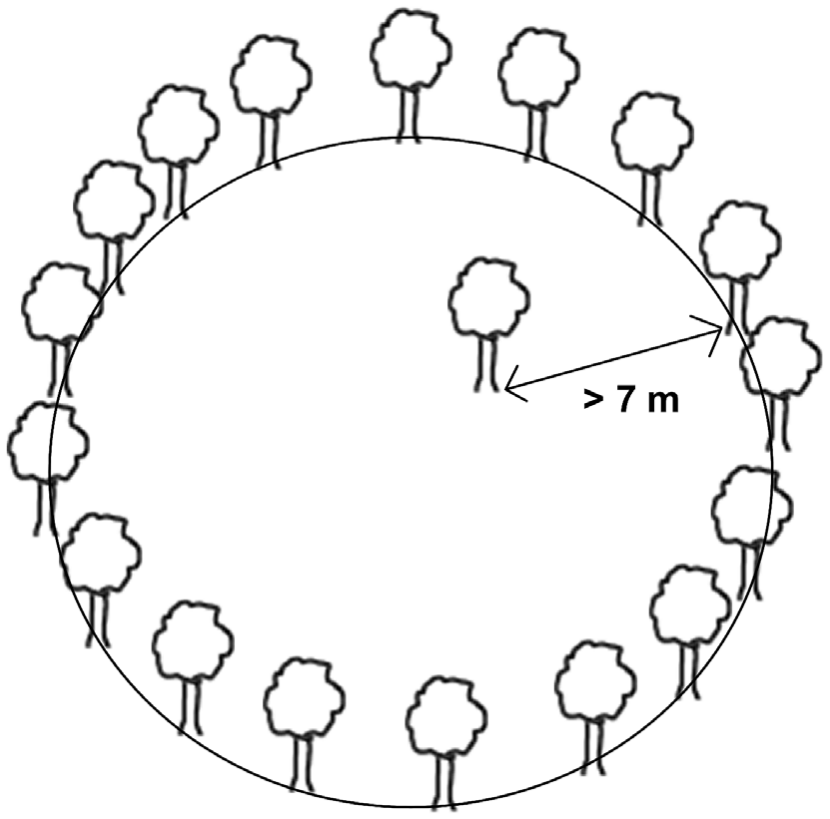
Definition of locality and its separation from the forest matrix.

For the purpose of the study, we defined the locality of the forest opening as every open patch with less than 30% tree cover (i.e., there could be some individual trees inside the locality, but they were isolated from the forest matrix by at least 7 m of the opening), which was isolated from other patches by at least 20 m of forest. The localities were thus delimited by the trunks of trees of forest matrix growing around each patch (Figure 2). The border between the grassland and the forest was always abrupt without a transient zone.

The occurrence of localities in the area is defined by a combination of summit and river phenomenon [50], by exposition, climate, geological and soil conditions and by human activities in the past.

### Data Collection

#### Field data collection

In the field, we recorded all vascular plant species at each locality. We surveyed each locality twice a year (from April to August 2005–2007) to include both the spring and summer plant flowering period. We used the plant nomenclature defined by Kubat et al. [51].

During the field survey, we observed that some species were growing not only in the forest openings but also in the surrounding forests. To study the effects of isolation and habitat size on species richness, we thus decided to exclude these species from the analyses. This was important for the species occurring in the forests, as the localities are in fact not isolated. Studying the effects of isolation on the forest species would thus not be sensible. To exclude these species, we generated a list of species growing both in the study localities and in the surrounding forests in the study region. The species that were included in the analysis belonged to the xerophilous vegetation units (*Asplenietea trichomanis, Festuco-Brometea, Sedo-Scleranthetea, Thlaspietea rotundifolii, Trifolio-Geranietea*) and to ruderal vegetation units (*Artemisietea vulgaris, Chenopodietea*), whereas the excluded species belonged to the vegetation of ancient dry acidophilous oak forests (*Quercion petraeae*, *Genisto germanicae-Quercion*). In this way, we excluded 34% of the 296 species recorded at the studied localities (Table S1). The tests not excluding these species provided similar results and are presented in Table S2.

At each locality, we recorded several abiotic conditions. First, we measured the height of the horizon from the center of each locality (at the ground level) in eight main directions using an inclinometer (in degrees) including the surrounding trees. We used these values to calculate the potential solar insolation of each locality (see below). We also recorded the proportion of each substrate category type on the localities (below noted as “substrate”) according to their soil depth into four categories: rock, shallow soil, deeper soil and scree. Rock was defined as a site with exposed rocks without any soil cover with several microhabitats, such as rock platforms and rock crevices, with specific conditions. Shallow soil was less than 10 cm deep and hosted vegetation of primitive soils and narrow-leaved dry grasslands. Sites with deeper soils (>10 cm) typically hosted vegetation of broad-leaved dry grasslands and dry herbaceous fringes. Scree was created by a moving substrate of relatively small stones. Although we originally measured the soil depth at the localities using a 2 mm diameter metal rod, the four categories (rock, shallow soil, deeper soil and scree) were in fact easy to distinguish visually. Therefore, we were able to differentiate and estimate the proportion of each substrate category type visually without exact mapping. To confirm our estimations, we measured the substrate depth by inserting a 2 mm diameter metal rod several times into the substrate at places with shallow and deeper soil at each locality with these substrate category types until it reached bedrock. To describe the substrate heterogeneity, we calculated the Shannon diversity index according to the proportions of substrate category types at each locality.

The study area is not the only area with forest openings in the region. To avoid the edge effects when calculating habitat isolation, we mapped all forest openings up to 500 m from the edge of the study area and used these only to calculate habitat isolation (see below).

#### Preparation of historical data

To assess the historical distribution of forest openings, we used aerial photographs of the study region from 1938, 1973, 1988 and 2000, which were provided by the Military and Hydrometeorological Institute of the Czech Republic. To input the historical forest openings into the GIS and calculate their geometry, we first rectified aerial photographs using PCI Geomatics 10.0 [52]. We used current orthophoto maps that provide accurate positions and cadastral maps that provide information about altitude to set control points. We used the same points from several aerial photographs of the same age as the tie points. Both types of points served to place aerial photographs into a system of coordinates. Then, we created a digital terrain model using a digital contour line map (with 2 m between contour lines and a raster cell size of 5 m×5 m), which we used to create historical orthophoto maps from the aerial photographs.

We classified the historical orthophoto maps using program Definiens [53]. This program can classify landscape structure based on the pixel value and on the structure and texture of classified subjects. Therefore, it is suitable for classifying black-and-white aerial photographs. We classified the historical landscape structure of the study region into two categories: forest and tree-less area. The relatively bad quality of some of the aerial photographs precluded the resolution of more categories. We manually verified and corrected the identified tree-less areas according to present-day conditions and our own field experience. We excluded all tree-less areas in positions unlikely hosting dry grasslands (e.g., a meadow in the valley next to a stream). From the old maps, we also excluded regularly shaped localities (i.e., squared), as these were likely to be artificial clearings that were subsequently reforested. The remaining tree-less areas were considered to represent the localities of forest openings.

In the same way, we also classified the current landscape structure of the study region using current orthophoto maps (2007) available from Geoportal Cenia (http://geoportal.cenia.cz). Additionally, we corrected the distribution and shape of all current localities according to our own field experiences because we thoroughly mapped them.

#### Landscape structure and local habitat conditions

Using GIS (ArcGIS 9.2 [54]), we calculated the geometry for each current locality: area, distance between localities and their isolation. Due to a high topographic heterogeneity, we calculated the surface area for each locality according to a digital elevation model of the terrain (created using a digital contour line map with a 2 m distance between contour lines and a raster cell size of 5 m×5 m and orthophoto maps). The isolation was calculated using a formula provided by Tremlova and Munzbergova [55]:
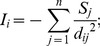
where *I_i_* represents the isolation of the *i*-locality, *j* represents all other localities in the circuit of 500 meters around the *i*-locality, *n* is the total number of localities in the circuit of 500 meters, *S_j_* is the area of *j*-locality (in m^2^), *d_ij_* is the distance between locality *i* and all other localities *j* (measured in m, as distance between locality centers because the localities do not exhibit an elongated shape). We considered the circuit of 500 meters because this distance was the best according to a sensitivity analysis of different distances (100 m, 200 m, 300 m, 500 m and 1000 m). Lobel et al. [24] also identified a circuit of 500 meters as the best for calculating isolation. The value of *I_i_* increases with increasing isolation of the locality. We also calculated the isolation of each current locality from past localities in each time period separately using the same formula.

According to the digital elevation terrain model (see above), we calculated several topographic heterogeneity parameters for each locality: slope and aspect using GIS and topographic wetness index (TWI) using SAGA GIS (http://www.saga-gis.org). The median, minimum, maximum and standard deviation values of the slope were used as a basis for subsequent analyses. We also used the slope and aspect values and the above mentioned values for the horizon height to calculate the potential direct solar insolation (PDSI) of each locality on the 21^st^ day of every month from December to June [56]. We also considered calculating the heat load index [57] for the localities but did not include it at the end, as it highly correlated with PDSI, and the algorithm for calculating these two values is extremely similar. Moreover, PDSI takes shading by adjacent topography and the surrounding trees into account and is therefore a better predictor of species richness especially at relatively small localities such as in our study system [57]. Moreover, Turtureanu and Dengler [25] observed a close correlation between the heat load index and the locality slope, and the slope was also included in our model. For calculating the TWI, we first calculated the slope (using a local morphometry function with a fit 2 degree polynom) and specific catchment area (using a parallel processing function with multiple flow direction method and number one convergence) according to the digital elevation terrain model (DEM). According to these parameters, we calculated the topographic wetness index (TWI) for each locality (with an area conversion of 1/cell size) (see [58] for details).

According to the geological map of the area (1∶50,000) [59], we classified the geological conditions of each locality into two categories: andesites and dacites.

Other parameters we calculated using the GIS served to describe a historical landscape structure: the historical area of each current locality (expressed as the area of historical localities around each current locality in a 30 m circuit, also calculated as surface area), historical isolation (see above) and continuity of localities (expressed as the percentage of overlap of each current locality with past locality in each time period separately). It is also possible that one historically large locality was divided into several smaller localities in the present, which is also represented by the parameter of the historical area of the locality (area 1938, area 1973, area 1988 and area 2000).

As supplementary information, even though we did not obtain direct measurements of some other habitat conditions, we calculated Ellenberg indicator values [60] for light, temperature, soil reaction pH and nutrient at each locality using the species compositions at the localities. Numerous studies have used this approach for indirectly estimating the habitat conditions [61]–[64].

### Statistical Analysis

The statistical analyses were performed using S-plus 4.6 [65] and Statistica 7.0 [66]. The dependent variable, the number of species, was normally distributed, and so no transformation of the data was needed.

To reduce the number of independent PDSI and slope variables, we used a pair-wise correlation matrix (Statistica 7.0 [66]) and selected the least correlated ones for use in subsequent analyses (for PDSI – median value for December and June; for slope – median and maximum value). To assess the relationship between all variables and their categories, we also calculated a pair-wise correlation matrix (Table S3) in Statistica 7.0 [66].

To take into account the linear spatial trends (spatial auto-correlation) of parameters describing localities across the study area, we used variation partitioning according to Borcard et al. [67] and Legendre and Legendre [68], which enabled us to separate the pure spatial component from the pure environmental component and their shared contribution. To identify complex spatial trends, seven derived geographical variables were constructed by including all quadratic and cubic combinations of x and y as suggested by Borcard et al. [67]: x, y, x^2^, xy, y^2^, x^3^, x^2^y, xy^2^, y^3^. We used the values selected in the stepwise regression (x, y, xy) as covariates in all subsequent tests to remove the effect of spatial position of the localities (spatial auto-correlation) because we wanted to study only the pure effect of the environment and not of the spatial component.

The tests of the effects of all the independent variables (Table 1) on species richness were performed in three steps. First, we tested the effect of each variable on species richness separately without any covariates, which represents the fraction of variation explained by non-spatial environmental variation and spatially structured environmental variation together (e.g., shared contribution of environmental and spatial variation). Second, we used the geographical coordinates of the center of each locality (those selected in the stepwise regression) as covariates and tested the effect of each independent variable separately, which represents the fraction of variation explained by non-spatial environmental variation [67], [68]. Finally, to obtain the pure effect of each variable after removing spatially structured environmental variation and any other shared variation with all other parameters, we used geographical coordinates and all significant factors from the second analyses as covariates (according to [67] and [69]). We used an analysis of variance (ANOVA) with type III sum of squares (S-plus 4.6) to identify a real effect of particular factors without the effect of all other factors. In the case of categorical variables with more than one category (substrate) and variables with multiple levels (slope, PDSI), we analyzed these categories or levels together using the difference between the models with and without all of the categories or levels. The means, medians and ranges of each predictor and dependent variable per locality are presented in Table 2.

**Table 1:** All studied variables enrolled in subsequent analyses.

Group of variables	Description
Coordinates	x, y, xy
Substrate	Four categories: Rock, shallow soil, deeper soil, scree (%)
Substrate heterogeneity	Shannon diversity index of substrate types
Slope	Slope (median+max) = median and maximum values of slope;Slope - STD = standard deviation values of slope
PDSI	PDSI (Dec+June) = median values of PDSI in December and June;PDSI - STD (Dec+June) = standard deviation values of PDSI in December and June
Geology	Andesites or dacites
TWI	Topographic wetness index
Area	log area: historical (1938, 1973, 1988, 2000) and current (2007)
Isolation	log isolation: historical (1938, 1973, 1988, 2000) and current (2007)

**Table 2:** Means, medians, minima and maxima of species numbers (per locality) and the independent variables.

Variable		Mean	Median	Min	Max	Units
Total species number		58.30	60.5	4	153	
Number of dry grasslandopenings species		37.58	37.5	1	115	
Local habitat	Substrate - rock	0.34	0.25	0	1	proportion
conditions	Substrate - shallow soil	0.30	0.1	0	1	proportion
	Substrate - scree	0.22	0	0	1	proportion
	Substrate - deeper soil	0.14	0	0	1	proportion
	Substrate heterogeneity	0.47	0.50	0	1.28	
	Slope - median	27.32	28	8	41	degrees
	Slope - max	30.14	30	13	48	degrees
	Slope - STD	1.82	1.24	0	9.52	degrees
	PDSI - median - Dec	0.92	0.54	0	4.89	
	PDSI - median - June	5.87	6.22	0	8.48	
	PDSI - STD - Dec	0.33	0.20	0	1.22	
	PDSI - STD - June	5.47	5.57	0	8.14	
	Geology	0.83	1	0	1	
	TWI	4.76	4.52	3.27	10.48	
	Ellenberg - L	7.36	7.38	6.71	8	
	Ellenberg - T	5.91	5.89	5.29	7	
	Ellenberg - R	6.18	6.29	3.50	8	
	Ellenberg - N	3.16	3.15	1.86	4.50	
Current landscape	Log area 2007	2.50	2.46	1.42	4.10	m^2^
structure	Log isolation 2007	0.15	0.14	−0.47	1.28	
Historical landscape	Log area 1938	1.43	1.80	0	3.16	m^2^
structure	Log area 1973	2.29	2.71	0	3.46	m^2^
	Log area 1988	2.08	2.50	−1.20	3.90	m^2^
	Log area 2000	2.56	2.63	0	3.47	m^2^
	Log isolation 1938	0.33	0.28	−1.10	5	
	Log isolation 1973	0.06	−0.03	−0.69	5	
	Log isolation 1988	0.07	0.06	−0.69	5	
	Log isolation 2000	0.15	0.15	−0.49	1.29	

For the abbreviation explanations, see Table 1.

For assessing the species-area relationship, we compared the fit provided by two alternative functions, a logarithmic function and a power function, using SPSS [70]. The test revealed that the logarithmic function explained a higher amount of variation in our data (R^2^ = 0.36) than the power function (R^2^ = 0.22). We thus decided to use the logarithmic function in the following analyses.

Alternatively, it is also possible to test the effect of studied factors on species richness using a multimodel inference analysis based on AIC [71], which would alleviate the problem of testing many partially correlated variables using p-values. The comparison of models based on AIC yielded extremely similar results to our first approach, and thus, only the results based on the p-values are presented.

To assess the relative importance of the three groups of factors (current landscape structure, historical landscape structure and habitat conditions) for species richness, we analyzed the effect of each of these three groups of factors alone. We also tested the effect of each of these groups of factors after using the other two groups of factors as covariates. Because each group contained too many independent variables, we chose only those factors that were significant when testing their independent effect on species richness using only coordinates as covariates. We used variance partitioning (according to [69]) to calculate the proportion of variance explained by each group of factors. We expressed the portion of explained variance as a relative part of the total explained variance.

## Results

### Changes in Landscape Structure

**Figure 3:**
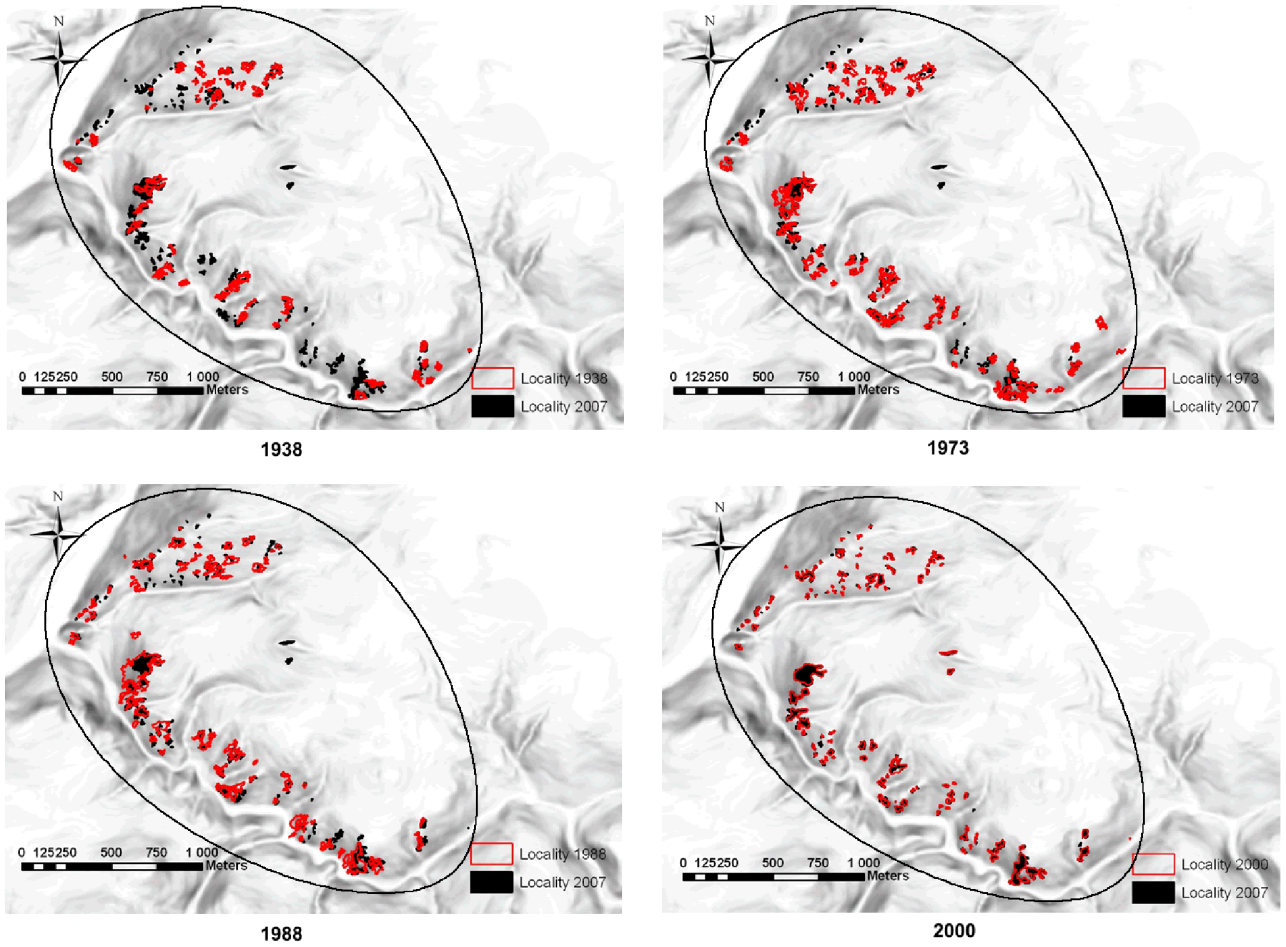
Distribution of forest openings in 1938, 1973, 1988, 2000 (red) in comparison with the current distribution –2007 (black). The line defines the border of our study area.

During the 20^th^ century and at the beginning of the 21^st^ century, the landscape structure of the study region changed. Specifically, there were fewer patches of forest openings in the past, but they were larger and more interconnected in comparison with the current landscape (Table 3). Today, we can observe a gradual reduction in the area of forest openings and an increase in their isolation (Figure 3).

**Table 3:** Numbers and mean areas of forest openings in each studied period in the past and in the present.

Year	Number of forest openings	Mean area of forest openings (m^2^)
1938	66	650
1973	105	840
1988	89	1080
2000	103	720
2007	110	635

### Correlations between Parameters

We identified many strong correlations within the group of historical parameters. The highest correlation was between the continuity of localities and their historical area. We thus decided to include only the historical area parameter into subsequent analyses because of its higher predictive power. There was no significant correlation within the group of current parameters and within the group of habitat conditions and between all of the three groups of parameters. The current area and isolation of the localities were also not significantly correlated, whereas the historical area and isolation were slightly correlated (Table S3).

### The Effect of Studied Factors on Species Richness

**Figure 4:**
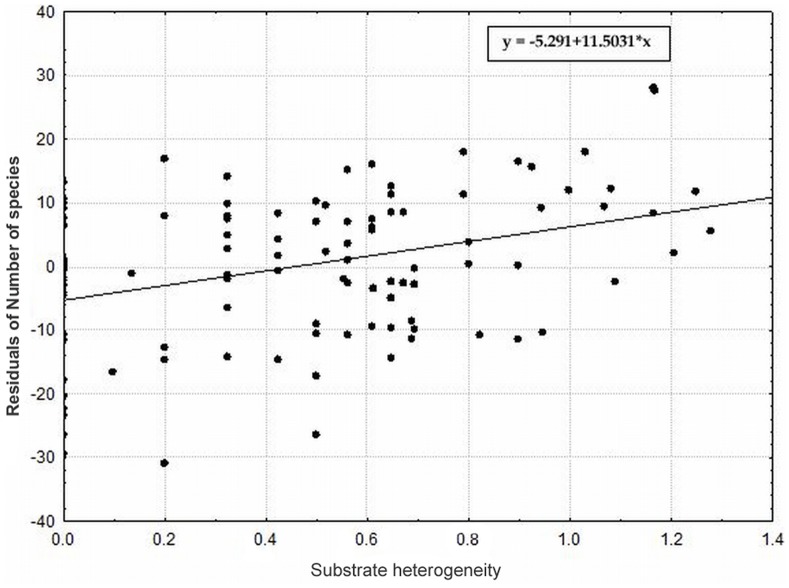
The effect of the substrate heterogeneity on species richness. The effect of the substrate heterogeneity at localities (Shannon diversity index of substrate types – rock, shallow soil, scree and deeper soil) on species richness (depicted as residuals of number of species with coordinates and all significant factors from the second analyses as covariates). P<0.0001; r = 0.255.

**Figure 5:**
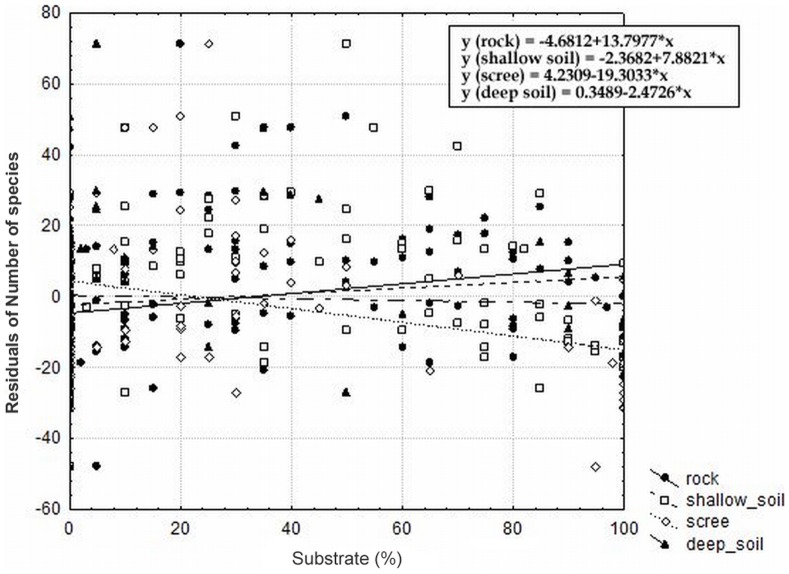
The effect of the substrate on species richness. The effect of the substrate in sense of the proportion of different types of substrate (rock, shallow soil, scree and deeper soil) on species richness (depicted as residuals of number of species with coordinates as covariates). P = 0.0054; r = 0.245 for rock, p = 0.154; r = 0.128 for shallow soil, p = 0.0003; r = −0.314 for scree and p = 0.6813; r = −0.037 for deeper soil.

**Figure 6:**
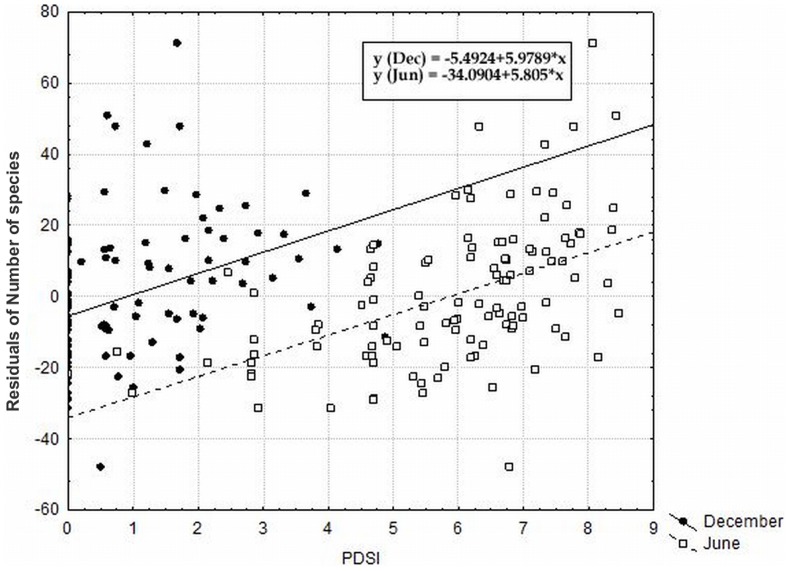
The effect of PDSI on species richness. The effect of potential direct solar insolation (PDSI) on December 21st and June 21st on species richness (depicted as residuals of number of species with coordinates as covariates). P<0.0001; r = 0.360 for December and p<0.0001; r = 0.510 for June.

The local habitat conditions, especially the substrate and substrate heterogeneity, exhibit strong significant effects on the species richness even after removing the effect of other significant independent parameters (Table 4). The substrate heterogeneity was the most important overall predictor of species richness, where it alone accounted for 40% of the total variation and 6.5% of the variation after removing all other significant factors. The most heterogeneous localities thus hosted the highest number of dry grassland species (Figure 4). Additionally, the substrate, in sense of the proportion of different substrate types, strongly affected the species richness (Figure 5). Localities represented by more types of substrate possessed the highest number of species. The number of species increased with increasing proportions of rock and shallow soil on the locality and decreased with increasing scree proportions. We also observed a higher richness on localities with a higher PDSI (Figure 6), a higher slope and with a higher standard deviation of PDSI and slope (Table 4). Their effects, however, disappeared when taking all of the other significant independent factors as covariates. The geology and topographic wetness index did not have a significant effect on species richness (Table 4). The Ellenberg indicator values suggest that only the light (L) values had a significant positive effect on species richness (p = 0.015; R^2^ = 0.047), which is indicative of more dry-grassland species on more open habitats. Other indicator values (for temperature, soil reaction pH and nutrient) did not significantly affect the species richness in our system (p = 0.989 for temperature, p = 0.270 for soil reaction pH and p = 0.855 for nutrient).

**Table 4:** The effect of studied factors on species richness.

		D.F.	Withoutcovariates	Covariates		Direction ofsignificance
				Coordinates (15.69%)	All significant factors (77.84%)	
Local	Substrate	3	14.15%	13.46%	3.17%	+/− *
habitat	Substrate heterogeneity	1	39.90%	32.38%	6.51%	+
conditions	Slope (median+max)	2	15.35%	13.31%	n.s.	+
	Slope - STD	1	12.35%	11.09%	n.s.	+
	PDSI (Dec+June)	2	35.52%	27.07%	n.s.	+
	PDSI - STD (Dec+June)	2	35.80%	26.26%	n.s.	+
	Geology	1	4.23%	n.s.	–	
	TWI	1	4.59%	n.s.	–	+
Current	Area 2007	1	36.03%	28.40%	1.18%	+
landsc.str.	Isolation 2007	1	n.s.	–	–	
Historical	Area 1938	1	19.04%	19.37%	2.80%	+
landscape	Area 1973	1	13.30%	8.28%	n.s.	+
structure	Area 1988	1	17.68%	9.92%	1.23%	+
	Area 2000	1	22.21%	16.83%	n.s.	+
	Isolation 1938	1	6.65%	5.40%	2.20%	–
	Isolation 1973	1	n.s.	–	–	
	Isolation 1988	1	n.s.	–	–	
	Isolation 2000	1	n.s.	–	–	

The effect of local habitat conditions, current and historical landscape structure on species richness and the direction of the effect (+/−). The amount of explained variance by the single independent variables with different covariates is presented; n.s. is not significant (p>0.05), – not tested because previously not significant. Df error = 108 (respectively, 106 for substrate and 107 for slope and for PDSI) for all factors significant without covariates, Df error = 105 (respectively, 103 for substrate and 104 for slope and for PDSI) for all factors significant when using coordinates as covariates and Df error = 87 (respectively, 85 for substrate and 86 for slope and for PDSI) for all factors significant when using coordinates and all significant factors from the first analyses as covariates. For the abbreviation explanations, see Table 1.

*(+) for rock, (–) for scree.

**Figure 7:**
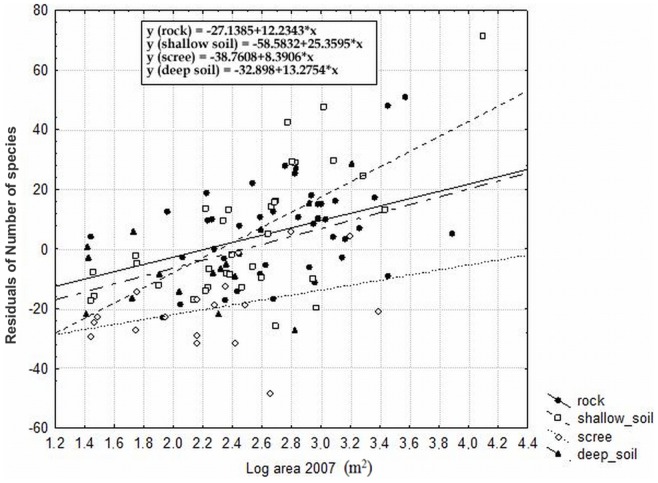
The effect of current area and prevailing type of substrate on species richness. (depicted as residuals of number of species with coordinates as covariates). P<0.0001; r = 0.533 for area and p = 0.0004; r = 0.367 for type of substrate.

Among factors describing the current landscape structure, only the current area of localities had significant and strong effect on the number of species. It was also the second most important overall predictor of species richness, where it alone explained 36% of the total variation in the dataset (Figure 7, Table 4). The effect of the current area was significant also after removing the effect of habitat heterogeneity (substrate, substrate heterogeneity, slope and PDSI and their standard deviations) and historical parameters of landscape structure (area in each studied periods and isolation in 1938), indicating a significant but much weaker (1.2% of explained variation) effect of the area (Table 4). In contrast to strong significant effects of the area, habitat isolation did not significantly affect the species richness (Table 4).

**Figure 8:**
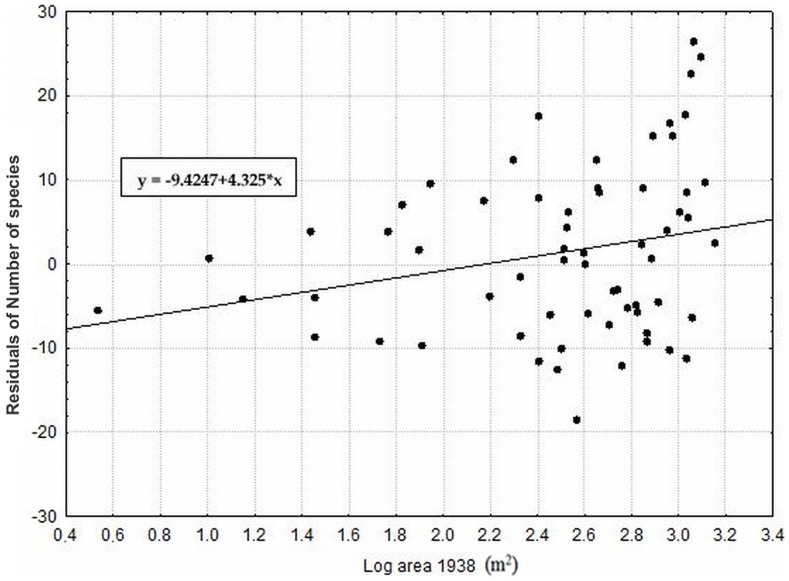
The effect of historical area of the locality in 1938 on species richness. (depicted as residuals of number of species with coordinates and all significant factors from the second analyses as covariates). P = 0.0012; r = 0.167.

**Figure 9:**
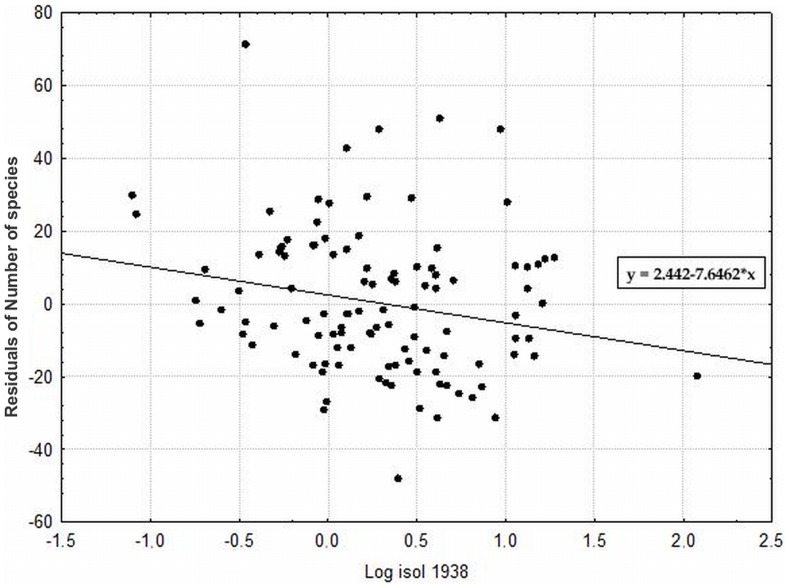
The effect of historical isolation in 1938 on species richness. (depicted as residuals of number of species with coordinates as covariates). The outlier values for the number of species have localities with currently higher species richness regardless of their isolation in the past, and the outlier value in isolation has a locality that was much more isolated from other localities in the past and also in the present. P = 0.0325; r = −0.232.

Among factors describing the historical landscape structure, the highest species richness was observed on localities with a larger area in 1938 (Figure 8), with a low isolation in 1938 (Figure 9) and with a larger area in 1988. These factors were significant even after removing the effects of all of the other significant factors (Table 4), suggesting that the effects of these factors are partly independent on the effect of the current area and habitat heterogeneity. Moreover, this pure effect of each of these historical factors explained even more variation in species richness than the pure effect of current habitat area (i.e., pure effect of these variables after removing the effects of all of the other significant factors from the second step of analyses). Additionally, the localities with a larger area in 1973 and 2000 exhibited a higher species richness when tested separately (Table 4).

**Figure 10:**
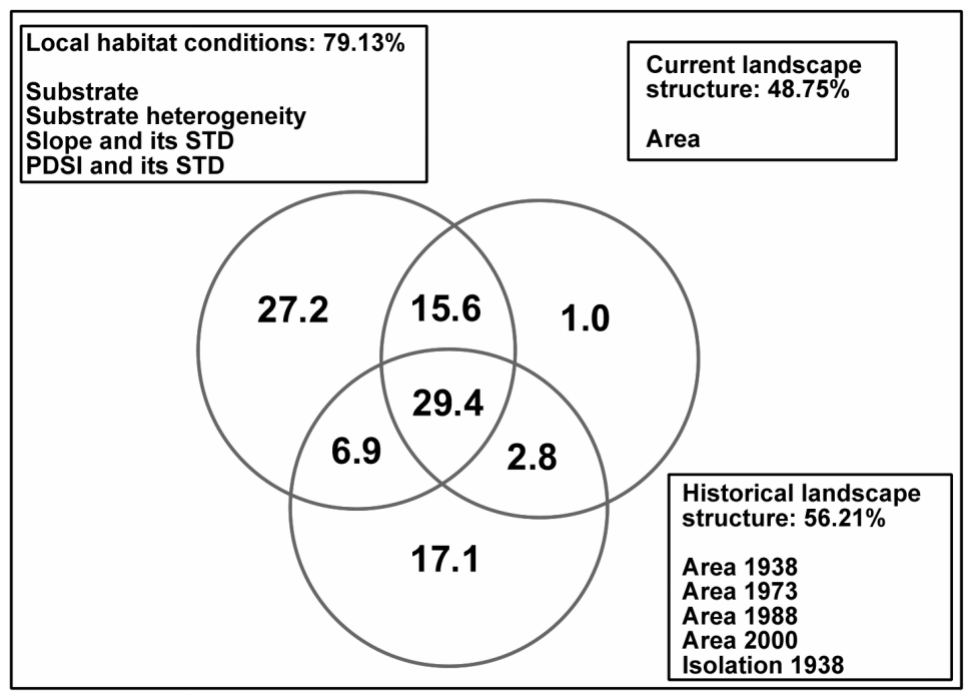
Variation explained by the local habitat condition, current landscape structure and historical landscape structure. For each group of factors, the significant predictors are ranked from the predictor with the highest explanatory power.

Altogether, the three groups of factors explained 73.9% of the species richness variance. Local habitat conditions explained 79.1% of the variation that could be explained by all of the factors together. The current landscape structure explained 48.8% of this variation, and the historical landscape structure explained 56.2% of this variation (Figure 10). The variance explained by each of the groups largely overlaps, with over 30% of the variation being attributable to at least two groups of factors.

## Discussion

The results of this study demonstrated that the current species richness of forest openings was significantly affected by all three studied groups of variables: the local habitat conditions and the landscape structure in the present and in the past. Each of these three groups explained different but important deal of species richness variance. Local habitat conditions have the strongest effect on species richness, followed by historical landscape structure. Current landscape structure has the weakest effect. The substrate heterogeneity seems to be the most important overall factor. From historical parameters the area of habitats in 1938 was the most important and from current parameters it was the current area of habitats.

### Changes in Landscape Structure

During the last several decades, some important changes occurred in the landscape structure of the study region, which had strong effects on the occurrence of dry grassland species at the localities. At first, from 1938 to 1988, the locality area increased by 124%. Since 1988, the area has decreased by 27%. The changes in the landscape structure of the study region were thus substantially different and the reduction of the locality area was not so dramatic in comparison with other studies on semi-natural dry grasslands performed in Belgium [23] or Sweden [3], where an area decline of approximately 90% was observed. This is because the Křivoklátsko region fulfilled a specific function in the past (local forests were used for hunting by the nobility especially in the Middle Ages). The main impact of human activity in the study region occurred much later (in the 18^th^ and 19^th^ centuries) and was not so intensive as in the other regions, which led to a well-preserved nature with large forest coverage [48]. The changes in the locality area and connectivity during the studied period could also be attributed to an outbreak and subsequent gradual reduction of hoofed game (especially mouflons) in the region. The mouflons were distributed in the region after 1938, then their populations gradually increased to a peak in the 1970s and 1980s. Since then, their numbers gradually decreased [46], [48], corresponding to changes in landscape structure (Figure 3, personal observation but not tested). The occurrence of mouflons could have a strong positive effect on the persistence of forest openings because if game browsing stops completely, gradual encroachment of shrubs and thus a temporary increase and then a consequential decrease in the species richness would occur. However, the high numbers of mouflons could negatively affect the survival of dry grassland species due to strong eutrophication of some places ([47]; personal observation). In our analysis the number of plant species was, however, not significantly affected by nutrient availability at the localities (expressed by Ellenberg indicator value for N), but it negatively responded to low light availability at the locality (expressed by an Ellenberg indicator value for L). Similarly, Turtureanu and Dengler [25] observed an important positive effect of canopy openness (and thus light availability) on species diversity in Carpathian forest openings. Nature conservation management should thus balance both mechanisms [47]: hunting of mouflons (it was partly practiced) and removal of young trees and shrubs colonizing grassland areas (this type of management was not practiced).

### Determinants of Species Richness

Each studied variable group (the local habitat conditions and landscape structure in the present and in the past) had a different effect on the species richness, and their relative importance also slightly differed. Similar to Adriaens et al. [23], who explored the effects of the same three groups of variables on species richness of semi-natural grasslands, we identified a strong significant effect of the current landscape structure and local habitat conditions. However, contrary to their study, we also identified a strong significant effect of the historical landscape structure on species richness. Lindborg and Eriksson [43] and Helm et al. [5] also observed a significant effect of the past landscape structure but not of current factors on species diversity. Only the study performed in forests [8] observed a significant effect of all three groups of variables on species richness.

The most important overall predictor of richness of dry grassland species was the substrate heterogeneity followed by the current locality area. The number of species increased with increasing locality area and thus confirmed the positive species-area relationship [15], [16], [32]. A larger area per se supports more species. In agreement with Ricklefs and Lovette [19], we also observed an indirect effect of area on species richness via increasing heterogeneity of the larger localities (according to the substrate heterogeneity and the proportion of each substrate type at the localities (i.e., “substrate”)). More heterogeneous localities and localities with a prevalence of rocks and shallow soil harbored more species than localities with deeper soil because the rocks and shallow soil provided numerous different microhabitats with specific conditions and thus enabled coexistence of many dry grassland species. In contrast to our study, Dornbush and Wilsey [72] and Oberndorfer and Lundholm [73] observed increasing richness with increasing soil depth in a tall grass prairie and in heathlands, but they performed their studies on a plot-scale and not on a patch-scale as we did. Conversely, Adriaens et al. [23] did not detect any effect of soil depth on species richness in semi-natural grasslands. In addition, a higher range of potential direct solar insolation and slope (standard deviation of these parameters) provided higher habitat heterogeneity and thus such localities host more species.

Species richness also increased with increasing PDSI and slope, which agrees with Adriaens et al. [23]. Localities with higher PDSI and slope values are typically occupied by more stress tolerant species, i.e., the species typical for rocks and dry grasslands (such as *Sedum acre, S. album, S. reflexum, Scleranthus perennis, Potentilla arenaria, Aurinia saxatilis*). Conversely, the localities with lower values of these factors are likely to be occupied by more competitive species (such as *Bromus sterilis, Carduus nutans, Echium vulgare, Geranium columbinum*) and many weaker competitors (such as *Arabidopsis thaliana, Alyssum alyssoides, Erophyla verna*) are thus excluded from the sites.

In contrast to Huerta-Martinez and Garcia-Moya [74] and Chylova and Munzbergova [26], we did not observe an effect of geology on species richness in our study because we only investigated two extremely similar types of geology (andesites and dacites), which possesses a similar pH, and thus, relatively similar soil types developed at these substrates. This most likely also accounted for the small range of values of the soil pH (expressed by the Ellenberg indicator value for soil reaction pH) and can explain why we did not find any effect of soil pH on species richness although other studies with more heterogeneous habitat conditions found soil pH to be extremely important [24], [27].

We also did not observe an effect of the topographic wetness index (TWI) on species richness most likely due to the small variance of TWI values, in contrast to Kopecky and Cizkova [58], who studied its effect on species composition.

Another important factor driving species richness of the habitats in fragmented landscapes could be their isolation. In contrast to strong significant effects of area, current habitat isolation did not significantly affect the species richness in the study region. Significant effects of isolation are commonly reported when studying oceanic islands due to much higher distances between islands than between habitat patches in the mainland (e.g., [32], [75]) and because the real islands are isolated for much longer and their diversity already reached equilibrium [16], [28]. In contrast to our study, many previous studies (e.g., [8], [55], [76]) observed a significant effect of habitat isolation even in mainland habitats at a spatial scale similar to that studied here.

The absence of a significant effect of habitat isolation in our study system could be explained by a relatively small distance between localities, which was not enough to affect the colonization process of species or affecting only some species not adapted to long-distance dispersal (such as *Asperula cynanchica, Aurinia saxatilis, Centaurea scabiosa, Helianthemum grandiflorum* agg., *Securigera varia*) [12], [28]. However, well dispersed species, usually with a partly ruderal strategy (such as *Bromus sterilis, Carduus nutans, Hieracium pilosella, Senecio viscosus, Setaria viridis, Taraxacum* sect. *Erythrosperma*), may replace them and thus yield a similar number of species with different traits on localities that differ in isolation [22], [55]. High species richness at isolated localities can also be maintained due to high species survival since the periods when the locality has been more connected, indicating an extinction debt [10], [77]. Species can survive either in the aboveground vegetation (long-lived species) or in the seed bank [10], [76]–[78]. This can explain the fact that despite no significant effect of current isolation, species richness of forest openings was significantly affected by historical isolation, specifically isolation in 1938, in the study region. According to Lindborg and Eriksson [43], Helm et al. [5] and Krauss et al. [44], it is possible that in the more distant history, the effect of habitat isolation on species richness would have been much stronger. In their studies, they did not detect any effect of current landscape structure on plant species richness of semi-natural grasslands but did observe a strong effect of landscape structure in the 1950s and an even stronger effect in the 1900s and in the 1930s.

Among factors describing historical landscape structure, the locality areas was much more important in comparison with locality isolation, especially the areas in 1938 and in 1988 and partially areas in 1973 and 2000. The historical area of the localities strongly correlated with their continuity, and thus, the localities with larger areas in the past have longer uninterrupted development. In such localities, species-rich communities with many rare species well adapted to live in specific habitats may develop. These species are usually perennials that are good competitors but bad colonizers and are thus able to persist on continuous localities for a long time. Higher richness on localities with a large historical area also agrees with previous studies performed in semi-natural grasslands (e.g., [5], [42], [43]) and in forests (e.g., [6], [8], [44], [79]), documenting a delay in the species richness response to fragmentation and changes in the landscape structure. However, in contrast to the above mentioned studies, we also observed a strong effect of current area on species richness. As suggested by Oster et al. [80], such a significant effect of current habitat area in contrast to area in the past indicates that the system is relatively in equilibrium. This may be consistent with the fact that habitats in our study area changed relatively little compared to other areas due to a higher local management stability.

Because of the relatively small study region area, and thus a small range of predictors that could be encompassed, some abiotic factors did not significantly affect the species richness; nevertheless, many of them (mainly elevation, standard deviation of elevation, topographic heterogeneity, especially shape of relief, habitat diversity, and occasionally disturbances and potential vegetation at each locality) play extremely important roles, especially at larger, regional scales. For example, Kucera [45] and Valtr [81] demonstrated significant effects of these various factors on species diversity on a larger scale – the entire Protected Landscape Area and Biosphere Reserve Křivoklátsko in the Czech Republic.

## Supporting Information

Table S1 **All species of vascular plants recorded on study localities.** Included – species is included into analyses (1) or not (0), Frequency – number of localities with species presence.
(DOC)

Table S2 **The effect of studied factors on species richness of all species growing at localities (species of the xerophilous vegetation units as well as forest species).** The effect of local habitat conditions, current and historical landscape structure on species richness and the direction of the effect (+/−). The amount of explained variance by the single independent variables with different covariates is presented; n.s. is not significant (p>0.05), – not tested because previously not significant. Df error = 108 (respectively, 106 for substrate and 107 for slope and for PDSI) for all factors significant without covariates, Df error = 105 (respectively 103 for substrate and 104 for slope and for PDSI) for all factors significant when using coordinates as covariates and Df error = 87 (respectively, 85 for substrate and 86 for slope and for PDSI) for all factors significant when using coordinates and all significant factors from the first analyses as covariates. For the abbreviation explanations, see Table 1.
(DOC)

Table S3 **Pair wise correlation matrix between individual variables of the local habitat conditions and the past and current landscape structure.** For the abbreviation explanations, see Table 1.
(DOC)
