# Supplementary material for: Relative Importance of Current and Past Landscape Structure and Local Habitat Conditions for Plant Species Richness in Dry Grassland-Like Forest Openings
Source: PLoS One. 2014 May 8;9(5):e97110. doi: 10.1371/journal.pone.0097110 (PMC4014584; doi:10.1371/journal.pone.0097110)
Supplement: Table S1 — All species of vascular plants recorded on study localities. Included – species is included into analyses (1) or not (0), Frequency – number of localities with species presence. (DOC) [file pone.0097110.s001.doc]

**Table S1**

**All species of vascular plants recorded on study localities.** Included – species is included into analyses (1) or not (0), Frequency – number of localities with species presence.

|  | **Included** | **Frequency** |
| --- | --- | --- |
| *Acer campestre* | 0 | 8 |
| *Acinos arvensis* | 1 | 13 |
| *Agrostis capillaris* | 1 | 18 |
| *Achillea millefolium (s.str.)* | 1 | 34 |
| *Ajuga genevensis* | 1 | 86 |
| *Alliaria petiolata* | 0 | 40 |
| *Allium oleraceum* | 1 | 37 |
| *Allium senescens subsp. montanum* | 1 | 24 |
| *Alyssum alyssoides* | 1 | 2 |
| *Alyssum montanum subsp. montanum* | 1 | 8 |
| *Anemone ranunculoides* | 0 | 4 |
| *Anchusa officinalis* | 1 | 32 |
| *Antennaria dioica* | 1 | 1 |
| *Anthemis arvensis* | 1 | 1 |
| *Anthemis tinctoria* | 1 | 4 |
| *Anthericum liliago* | 1 | 2 |
| *Anthetricum ramosum* | 0 | 47 |
| *Anthoxanthum odoratum* | 1 | 7 |
| *Anthriscus sylvestris* | 0 | 7 |
| *Arabidopsis thaliana* | 1 | 41 |
| *Arabis glabra* | 1 | 19 |
| *Arabis hirsuta* | 1 | 17 |
| *Arenaria serpyllifolia agg.* | 1 | 41 |
| *Artemisia absinthium* | 1 | 37 |
| *Artemisia campesris* | 1 | 1 |
| *Asarum europaeum* | 0 | 1 |
| *Asperula cynanchica* | 1 | 30 |
| *Asplenium ruta-muraria* | 1 | 7 |
| *Asplenium septentrionale* | 1 | 52 |
| *Asplenium trichomanes agg.* | 1 | 46 |
| *Astragalus glycyphyllos* | 1 | 27 |
| *Athyrium filix-femina* | 0 | 4 |
| *Atropa bella-donna* | 1 | 1 |
| *Aurinia saxatilis* | 1 | 7 |
| *Avenella flexuosa* | 0 | 28 |
| *Ballota nigra* | 0 | 15 |
| *Barbarea vulgaris* | 1 | 17 |
| *Betonica officinalis* | 0 | 18 |
| *Brachypodium pinnatum* | 1 | 70 |
| *Brachypodium sylvaticum* | 0 | 65 |
| *Bromus sterilis* | 1 | 14 |
| *Bupleurum falcatum* | 0 | 15 |
| *Calamagrostis arundinacea* | 0 | 42 |
| *Calamagrostis epigejos* | 0 | 15 |
| *Calluna vulgaris* | 1 | 5 |
| *Campanula persicifolia* | 0 | 31 |
| *Campanula rapunculoides* | 1 | 2 |
| *Campanula rotundifolia* | 1 | 26 |
| *Campanula trachelium* | 0 | 53 |
| *Capsella bursa-pastoris* | 1 | 6 |
| *Cardamine impatiens* | 0 | 6 |
| *Cardaminopsis arenosa* | 0 | 95 |
| *Cardaria draba* | 1 | 8 |
| *Carduus acanthoides* | 1 | 25 |
| *Carduus nutans* | 1 | 12 |
| *Carex caryophyllea* | 1 | 41 |
| *Carex digitata* | 0 | 3 |
| *Carex muricata agg.* | 1 | 87 |
| *Carex ovalis* | 1 | 4 |
| *Carex pilulifera* | 1 | 8 |
| *Carex praecox* | 1 | 10 |
| *Centaurea scabiosa* | 1 | 7 |
| *Centaurea stoebe* | 1 | 7 |
| *Cerastium arvense* | 1 | 32 |
| *Cerastium brachypetalum* | 1 | 30 |
| *Cerastium holosteoides* | 1 | 15 |
| *Cerinthe minor* | 1 | 2 |
| *Circaea lutetiana* | 0 | 1 |
| *Clinopodium vulgare* | 1 | 50 |
| *Convolvulus arvensis* | 1 | 14 |
| *Corydalis cava* | 0 | 2 |
| *Cotoneaster integerrimus* | 1 | 24 |
| *Crateagus sp.* | 0 | 63 |
| *Cruciata laevipes* | 1 | 3 |
| *Cuscuta europaea* | 0 | 1 |
| *Cynoglossum officinale* | 1 | 20 |
| *Cytisus nigricans* | 1 | 7 |
| *Dactylis glomerata* | 1 | 28 |
| *Dactylis polygama* | 0 | 11 |
| *Danthonia decumbens* | 1 | 2 |
| *Dianthus carthusianorum subsp. carthusianorum* | 1 | 25 |
| *Dictamnus albus* | 1 | 1 |
| *Digitalis grandiflora* | 1 | 22 |
| *Dryopteris filix-mas* | 0 | 11 |
| *Echium vulgare* | 1 | 46 |
| *Elymus caninus* | 0 | 1 |
| *Epilobium collinum* | 1 | 1 |
| *Epilobium montanum* | 0 | 13 |
| *Erodium cicutarium* | 1 | 6 |
| *Erophila verna* | 1 | 20 |
| *Euphorbia cyparissias* | 1 | 87 |
| *Euphrasia stricta* | 1 | 1 |
| *Fallopia convolvulus* | 0 | 77 |
| *Festuca heterophylla* | 0 | 6 |
| *Festuca ovina agg.* | 1 | 90 |
| *Festuca pallens* | 1 | 16 |
| *Festuca rubra agg.* | 1 | 10 |
| *Festuca rupicola* | 1 | 23 |
| *Festuca valesiaca* | 1 | 3 |
| *Ficaria verna* | 0 | 2 |
| *Filago arvensis* | 1 | 9 |
| *Fragaria moschata* | 0 | 18 |
| *Fragaria vesca* | 1 | 13 |
| *Fragaria viridis* | 1 | 86 |
| *Fumaria schleicheri* | 1 | 3 |
| *Gagea bohemica subsp. bohemica* | 1 | 1 |
| *Gagea villosa* | 1 | 6 |
| *Galeobdolon luteum* | 0 | 3 |
| *Galeopsis angustifolia* | 1 | 83 |
| *Galeopsis ladanum* | 0 | 43 |
| *Galium album agg.* | 1 | 52 |
| *Galium aparine* | 0 | 43 |
| *Galium glaucum* | 1 | 17 |
| *Galium odoratum* | 0 | 8 |
| *Galium pumilum* | 1 | 37 |
| *Galium verum* | 1 | 26 |
| *Genista germanica* | 1 | 25 |
| *Genista tinctoria* | 1 | 25 |
| *Geranium columbinum* | 1 | 38 |
| *Geranium pusillum* | 1 | 16 |
| *Geranium pyrenaicum* | 1 | 6 |
| *Geranium robertianum* | 0 | 31 |
| *Geranium sanguineum* | 1 | 4 |
| *Geum urbanum* | 0 | 73 |
| *Glechoma hederacea* | 0 | 8 |
| *Gnaphalium sylvaticum* | 1 | 6 |
| *Hedera helix* | 0 | 2 |
| *Helianthemum grandiflorum subsp. obscurum* | 1 | 3 |
| *Hepatica nobilis* | 0 | 9 |
| *Hieracium cymosum* | 1 | 19 |
| *Hieracium laevigatum* | 0 | 4 |
| *Hieracium lachenalii* | 0 | 3 |
| *Hieracium murorum* | 0 | 40 |
| *Hieracium pilosella* | 1 | 76 |
| *Hieracium sabaudum* | 0 | 5 |
| *Hieracium schmidtii* | 1 | 25 |
| *Holosteum umbellatum* | 1 | 4 |
| *Hylotelephium maximum* | 1 | 21 |
| *Hypericum montanum* | 0 | 7 |
| *Hypericum perforatum* | 1 | 98 |
| *Chaerophyllum temulum* | 0 | 2 |
| *Chelidonium majus* | 0 | 1 |
| *Chenopodium album (s.str.)* | 1 | 3 |
| *Chenopodium polyspermum* | 1 | 2 |
| *Impatiens noli-tangere* | 0 | 5 |
| *Impatiens parviflora* | 0 | 52 |
| *Inula conyzae* | 1 | 26 |
| *Jasione montana* | 1 | 25 |
| *Jovibarba globifera subsp. globifera* | 1 | 5 |
| *Juncus conglomeratus* | 0 | 1 |
| *Juniperus communis subsp. communis* | 1 | 2 |
| *Koeleria macrantha* | 1 | 7 |
| *Lactuca serriola* | 1 | 1 |
| *Lactuca viminea* | 1 | 1 |
| *Lamium album* | 0 | 1 |
| *Lamium amplexicaule* | 1 | 8 |
| *Lamium purpureum* | 0 | 17 |
| *Lappula squarrosa* | 1 | 2 |
| *Lapsana communis* | 0 | 45 |
| *Lathyrus niger* | 0 | 5 |
| *Lathyrus pratensis* | 1 | 1 |
| *Lathyrus vernus* | 0 | 3 |
| *Leucanthemum vulgare* | 1 | 2 |
| *Lilium martagon* | 0 | 3 |
| *Linaria vulgaris* | 1 | 25 |
| *Lithospermum purpurocaeruleum* | 1 | 5 |
| *Lotus corniculatus* | 1 | 26 |
| *Luzula campestris* | 1 | 16 |
| *Luzula luzuloides* | 0 | 45 |
| *Luzula multiflora* | 1 | 13 |
| *Lychnis viscaria* | 1 | 58 |
| *Lysimachia nummularia* | 0 | 2 |
| *Malus sylvestris* | 1 | 1 |
| *Matricaria discoidea* | 0 | 1 |
| *Medicago falcata* | 1 | 3 |
| *Medicago minima* | 1 | 1 |
| *Melica nutans* | 0 | 12 |
| *Melica picta* | 0 | 1 |
| *Melica transsilvanica* | 1 | 44 |
| *Melilotus officinalis* | 0 | 1 |
| *Melittis melissophyllum* | 1 | 3 |
| *Microrrhinum minus* | 1 | 2 |
| *Moehringia trinervia* | 0 | 18 |
| *Mycelis muralis* | 0 | 14 |
| *Myosotis arvensis* | 1 | 25 |
| *Myosotis ramosissima* | 1 | 23 |
| *Myosotis sparsiflora* | 1 | 13 |
| *Myosotis stricta* | 1 | 21 |
| *Myosotis sylvatica* | 0 | 23 |
| *Omphalodes scorpioides* | 0 | 3 |
| *Onopordum acanthium* | 1 | 3 |
| *Origanum vulgare* | 1 | 64 |
| *Orobanche alba agg.* | 1 | 2 |
| *Oxalis fontana* | 0 | 1 |
| *Papaver dubium agg.* | 1 | 5 |
| *Persicaria hydropiper* | 0 | 3 |
| *Persicaria lapathifolia* | 0 | 2 |
| *Petrorhagia prolifera* | 1 | 2 |
| *Phleum phleoides* | 1 | 40 |
| *Phyteuma spicatum* | 0 | 1 |
| *Pimpinella saxifraga* | 1 | 5 |
| *Plantago lanceolata* | 1 | 3 |
| *Plantago media* | 1 | 1 |
| *Poa angustifolia* | 1 | 57 |
| *Poa annua* | 0 | 4 |
| *Poa compressa* | 1 | 16 |
| *Poa nemoralis* | 0 | 87 |
| *Poa pratensis* | 1 | 13 |
| *Polygala vulgaris* | 1 | 1 |
| *Polygonatum odoratum* | 0 | 44 |
| *Polygonum aviculare* | 0 | 29 |
| *Polypodium vulgare* | 0 | 11 |
| *Potentilla arenaria* | 1 | 15 |
| *Potentilla argentea* | 1 | 54 |
| *Potentilla inclinata* | 1 | 2 |
| *Potentilla recta* | 1 | 14 |
| *Potentilla tabernaemontani* | 1 | 60 |
| *Primula veris* | 1 | 4 |
| *Prunus spinosa* | 1 | 74 |
| *Pseudolysimachion spicatum* | 1 | 6 |
| *Pulsatilla pratensis subsp. bohemica* | 1 | 4 |
| *Pyrethrum corymbosum* | 0 | 79 |
| *Pyrus pyraster* | 1 | 10 |
| *Ranunculus auricomus agg.* | 0 | 4 |
| *Ranunculus bulbosus* | 1 | 26 |
| *Ribes alpinum* | 0 | 3 |
| *Rosa sp.* | 0 | 95 |
| *Rubus sp.* | 0 | 27 |
| *Rumex acetosa* | 1 | 5 |
| *Rumex acetosella* | 1 | 57 |
| *Salvia nemorosa* | 1 | 2 |
| *Sambucus nigra* | 0 | 50 |
| *Sanguisorba minor* | 1 | 16 |
| *Scleranthus perennis* | 1 | 29 |
| *Securigera varia* | 1 | 67 |
| *Sedum acre* | 1 | 8 |
| *Sedum album* | 1 | 36 |
| *Sedum reflexum* | 1 | 24 |
| *Sedum sexangulare* | 1 | 56 |
| *Senecio viscosus* | 1 | 38 |
| *Seseli osseum* | 1 | 10 |
| *Sesleria caerulea* | 1 | 8 |
| *Setaria viridis* | 1 | 15 |
| *Silene nutans* | 0 | 81 |
| *Silene vulgaris* | 1 | 10 |
| *Solidago virgaurea subsp. virgaurea* | 1 | 8 |
| *Sonchus arvensis* | 1 | 5 |
| *Sorbus aria* | 1 | 12 |
| *Sorbus aucuparia* | 0 | 4 |
| *Stellaria holostea* | 0 | 56 |
| *Stellaria media* | 0 | 5 |
| *Stipa pennata* | 1 | 7 |
| *Symphytum officinale* | 0 | 1 |
| *Taraxacum sect. Erythrosperma* | 1 | 39 |
| *Taraxacum sect. Ruderalia* | 0 | 40 |
| *Teucrium botrys* | 1 | 16 |
| *Teucrium chamaedrys* | 1 | 4 |
| *Thlaspi arvense* | 0 | 2 |
| *Thlaspi perfoliatum* | 1 | 5 |
| *Thymus pulegioides subsp. chamaedrys* | 1 | 88 |
| *Torilis japonica* | 0 | 29 |
| *Trifolium alpestre* | 1 | 45 |
| *Trifolium arvense* | 1 | 21 |
| *Trifolium aureum* | 1 | 4 |
| *Trifolium campestre* | 1 | 11 |
| *Trifolium dubium* | 1 | 3 |
| *Trifolium medium* | 1 | 14 |
| *Trifolium repens* | 0 | 2 |
| *Triticum aestivum* | 1 | 1 |
| *Urtica dioica* | 0 | 52 |
| *Vaccinium myrtillus* | 0 | 4 |
| *Valerianella locusta* | 1 | 31 |
| *Verbascum densiflorum* | 1 | 22 |
| *Verbascum lychnitis* | 1 | 57 |
| *Veronica arvensis* | 1 | 10 |
| *Veronica dillenii* | 1 | 49 |
| *Veronica hederifolia* | 0 | 36 |
| *Veronica chamaedrys* | 0 | 74 |
| *Veronica officinalis* | 0 | 59 |
| *Veronica prostrata* | 1 | 10 |
| *Veronica sublobata* | 0 | 2 |
| *Veronica verna* | 1 | 18 |
| *Vicia angustifolia* | 1 | 22 |
| *Vicia hirsuta* | 1 | 35 |
| *Vicia pisiformis* | 0 | 1 |
| *Vicia sepium* | 0 | 4 |
| *Vicia tenuifolia* | 1 | 2 |
| *Vicia tetrasperma* | 1 | 30 |
| *Vincetoxicum hirundinaria* | 0 | 95 |
| *Viola arvensis* | 1 | 76 |
| *Viola collina* | 1 | 1 |
| *Viola hirta* | 1 | 8 |
| *Viola odorata* | 1 | 1 |
| *Viola reichenbachiana* | 0 | 24 |
| *Viola riviniana* | 1 | 33 |
| *Viola tricolor subsp. tricolor* | 0 | 30 |
