# Supplementary material for: Relative Importance of Current and Past Landscape Structure and Local Habitat Conditions for Plant Species Richness in Dry Grassland-Like Forest Openings
Source: PLoS One. 2014 May 8;9(5):e97110. doi: 10.1371/journal.pone.0097110 (PMC4014584; doi:10.1371/journal.pone.0097110)
Supplement: Table S2 — The effect of studied factors on species richness of all species growing at localities (species of the xerophilous vegetation units as well as forest species). The effect of local habitat conditions, current and historical landscape structure on species richness and the direction of the effect (+/−). The amount of explained variance by the single independent variables with different covariates is presented; n.s. is not significant (p>0.05), – not tested because previously not significant. Df error = 108 (respectively, 106 for substrate and 107 for slope and for PDSI) for all factors significant without covariates, Df error = 105 (respectively 103 for substrate and 104 for slope and for PDSI) for all factors significant when using coordinates as covariates and Df error = 87 (respectively, 85 for substrate and 86 for slope and for PDSI) for all factors significant when using coordinates and all significant factors from the first analyses as covariates. For the abbreviation explanations, see Table 1. (DOC) [file pone.0097110.s002.doc]

**Table S2**

**The effect of studied factors on species richness of all species growing at localities (species of the xerophilous vegetation units as well as forest species).** The effect of local habitat conditions, current and historical landscape structure on species richness and the direction of the effect (+/-). The amount of explained variance by the single independent variables with different covariates is presented; n.s. is not significant (p > 0.05), – not tested because previously not significant. Df error = 108 (respectively, 106 for substrate and 107 for slope and for PDSI) for all factors significant without covariates, Df error = 105 (respectively 103 for substrate and 104 for slope and for PDSI) for all factors significant when using coordinates as covariates and Df error = 87 (respectively, 85 for substrate and 86 for slope and for PDSI) for all factors significant when using coordinates and all significant factors from the first analyses as covariates. For the abbreviation explanations, see Table 1.

|  |  |  |  | Covariates | | Direction of significance |
| --- | --- | --- | --- | --- | --- | --- |
|  |  | D.F. | Without covariates | Coordinates (15.69%) | All significant factors (77.84%) |  |
| Local | Substrate | 3 | 8.96% | 9.70% | 3.29% | +/- |
| habitat | Substrate heterogeneity | 1 | 42.35% | 36.72% | 8.41% | + |
| conditions | Slope (median+max) | 2 | 12.16% | 13.19% | n.s. | + |
|  | Slope - STD | 1 | 11.90% | 11.98% | n.s. | + |
|  | PDSI (Dec+June) | 2 | 26.03% | 20.77% | n.s. | + |
|  | PDSI - STD (Dec+June) | 2 | 26.57% | 20.33% | 2.02 | + |
|  | Geology | 1 | n.s. | n.s. | – |  |
|  | TWI | 1 | n.s. | n.s. | – |  |
| Current | Area 2007 | 1 | 33.80% | 28.93% | 1.63% | + |
| land.str. | Isolation 2007 | 1 | n.s. | – | – |  |
| Historical | Area 1938 | 1 | 15.25% | 13.80% | 1.41% | + |
| landscape | Area 1973 | 1 | 10.20% | 5.58% | n.s. | + |
| structure | Area 1988 | 1 | 16.40% | 9.34% | 1.66% | + |
|  | Area 2000 | 1 | 23.57% | 18.73% | n.s. | + |
|  | Isolation 1938 | 1 | n.s. | – | – |  |
|  | Isolation 1973 | 1 | n.s. | – | – |  |
|  | Isolation 1988 | 1 | n.s. | – | – |  |
|  | Isolation 2000 | 1 | n.s. | – | – |  |

The idea of using only species growing exclusively at studied localities and nowhere else in the study region is that we want to study how locality isolation, among other parameters, affected local species diversity. To do such an analysis it is thus absolutely crucial to use only species for which the studied localities are the only possible localities in the area. The data shown in this Supporting Information S2 are thus partly wrong, and that is another reason for showing these only in the supplementary information.
