# Supplementary material for: Relative Importance of Current and Past Landscape Structure and Local Habitat Conditions for Plant Species Richness in Dry Grassland-Like Forest Openings
Source: PLoS One. 2014 May 8;9(5):e97110. doi: 10.1371/journal.pone.0097110 (PMC4014584; doi:10.1371/journal.pone.0097110)
Supplement: Table S3 — Pair wise correlation matrix between individual variables of the local habitat conditions and the past and current landscape structure. For the abbreviation explanations, see Table 1. (DOC) [file pone.0097110.s003.doc]

**Table S3**

Pair wise correlation matrix between individual variables of the local habitat conditions and the past and current landscape structure. For the abbreviation explanations, see Table 1.

|  |  | Substrate | | | |  | Slope | | | PDSI | | | |  | Geo |  |  |  |  |  |  |  |  |  |  |  |  |  |  |
| --- | --- | --- | --- | --- | --- | --- | --- | --- | --- | --- | --- | --- | --- | --- | --- | --- | --- | --- | --- | --- | --- | --- | --- | --- | --- | --- | --- | --- | --- |
|  |  | rock | shallow | scree | deep | Substr.  heterog | median | max | slope-STD | Dec | June | Dec-STD | Jun-STD | TWI | andesit | area07 | isol07 | area38 | area73 | area88 | area00 | isol38 | isol73 | isol88 | isol00 | cont38 | cont73 | cont88 | cont00 |
| Substrate | rock | 1.00 | **-0.32** | **-0.33** | **-0.36** | **0.23** | **0.49** | **0.56** | 0.09 | **0.36** | **0.41** | **0.37** | **0.40** | -0.12 | **-0.26** | **0.32** | 0.13 | -0.13 | -0.12 | -0.08 | 0.10 | **0.19** | 0.13 | -0.09 | 0.16 | -0.15 | -0.03 | 0.14 | 0.11 |
|  | shallow_soil |  | 1.00 | **-0.45** | **-0.29** | 0.15 | **-0.20** | -0.18 | 0.05 | -0.05 | 0.07 | -0.04 | 0.05 | -0.07 | -0.08 | -0.04 | 0.08 | 0.14 | -0.05 | 0.05 | -0.13 | -0.10 | 0.12 | 0.13 | 0.06 | 0.01 | -0.01 | 0.12 | **-0.24** |
|  | scree |  |  | 1.00 | **-0.25** | **-0.20** | 0.18 | 0.11 | -0.19 | -0.14 | **-0.23** | -0.15 | **-0.21** | **0.20** | **0.20** | -0.05 | -0.17 | -0.16 | **0.23** | -0.02 | 0.05 | 0.00 | **-0.26** | -0.09 | -0.16 | -0.17 | -0.02 | -0.04 | 0.04 |
|  | deep_soil |  |  |  | 1.00 | **-0.19** | **-0.51** | **-0.53** | 0.05 | -0.18 | **-0.27** | -0.19 | **-0.27** | -0.02 | 0.16 | **-0.24** | -0.05 | 0.15 | -0.07 | 0.04 | 0.00 | -0.10 | 0.00 | 0.04 | -0.07 | **0.34** | 0.07 | **-0.24** | 0.11 |
|  | Substr. heterog |  |  |  |  | 1.00 | 0.10 | **0.23** | **0.23** | **0.29** | **0.44** | **0.30** | **0.46** | -0.08 | -0.04 | **0.53** | 0.09 | **0.21** | **0.24** | **0.19** | **0.40** | -0.12 | -0.06 | -0.11 | 0.10 | 0.09 | **0.25** | **0.29** | **0.19** |
| Slope | median |  |  |  |  |  | 1.00 | **0.89** | **-0.22** | **0.39** | **0.32** | **0.35** | **0.36** | -0.10 | -0.08 | **0.27** | **-0.20** | -0.06 | **0.25** | -0.01 | 0.12 | 0.06 | -0.18 | **-0.36** | -0.17 | -0.08 | 0.14 | 0.13 | 0.01 |
|  | max |  |  |  |  |  |  | 1.00 | **0.20** | **0.47** | **0.43** | **0.40** | **0.48** | **-0.25** | -0.12 | **0.50** | -0.15 | -0.10 | **0.20** | -0.02 | **0.26** | 0.11 | -0.11 | **-0.36** | -0.09 | -0.15 | 0.11 | **0.19** | 0.10 |
|  | slope-STD |  |  |  |  |  |  |  | 1.00 | **0.21** | **0.28** | 0.12 | **0.30** | **-0.32** | -0.11 | **0.49** | 0.11 | 0.02 | -0.03 | 0.03 | **0.35** | 0.06 | 0.12 | 0.00 | 0.14 | -0.04 | -0.01 | 0.13 | **0.19** |
| PDSI | Dec |  |  |  |  |  |  |  |  | 1.00 | **0.57** | **0.95** | **0.63** | -0.13 | **-0.22** | **0.58** | -0.06 | **0.32** | **0.37** | 0.15 | **0.44** | **-0.28** | -0.12 | **-0.22** | -0.04 | **0.20** | **0.41** | **0.33** | **0.29** |
|  | June |  |  |  |  |  |  |  |  |  | 1.00 | **0.59** | **0.98** | -0.11 | -0.03 | **0.57** | -0.02 | **0.34** | **0.41** | **0.26** | **0.42** | **-0.23** | -0.03 | **-0.25** | 0.01 | 0.18 | **0.41** | **0.43** | **0.27** |
|  | Dec-STD |  |  |  |  |  |  |  |  |  |  | 1.00 | **0.64** | -0.14 | -0.14 | **0.58** | -0.09 | **0.32** | **0.38** | **0.24** | **0.43** | **-0.31** | -0.13 | **-0.23** | -0.06 | 0.18 | **0.41** | **0.37** | **0.28** |
|  | Jun-STD |  |  |  |  |  |  |  |  |  |  |  | 1.00 | -0.12 | -0.05 | **0.62** | -0.04 | **0.35** | **0.47** | **0.24** | **0.47** | **-0.25** | -0.06 | **-0.27** | -0.01 | **0.21** | **0.47** | **0.44** | **0.28** |
|  | TWI |  |  |  |  |  |  |  |  |  |  |  |  | 1.00 | -0.02 | **-0.21** | 0.06 | 0.18 | 0.17 | -0.04 | -0.17 | **-0.21** | -0.13 | 0.16 | 0.00 | 0.17 | 0.14 | -0.10 | -0.09 |
| Geo | andesites |  |  |  |  |  |  |  |  |  |  |  |  |  | 1.00 | -0.17 | -0.02 | 0.16 | 0.16 | **0.34** | -0.02 | **-0.28** | **-0.27** | -0.16 | -0.05 | **0.20** | **0.20** | **0.23** | 0.02 |
|  | area07 |  |  |  |  |  |  |  |  |  |  |  |  |  |  | 1.00 | -0.03 | 0.18 | **0.34** | **0.29** | **0.68** | -0.13 | -0.05 | **-0.23** | 0.00 | 0.06 | **0.30** | **0.37** | **0.45** |
|  | isol07 |  |  |  |  |  |  |  |  |  |  |  |  |  |  |  | 1.00 | 0.01 | **-0.22** | **-0.28** | -0.18 | **0.28** | **0.57** | **0.81** | **0.96** | 0.12 | -0.01 | -0.10 | 0.12 |
|  | area38 |  |  |  |  |  |  |  |  |  |  |  |  |  |  |  |  | 1.00 | **0.56** | **0.23** | **0.29** | **-0.69** | **-0.22** | -0.02 | -0.06 | **0.68** | **0.60** | 0.16 | **0.27** |
|  | area73 |  |  |  |  |  |  |  |  |  |  |  |  |  |  |  |  |  | 1.00 | **0.32** | **0.43** | **-0.54** | **-0.44** | **-0.26** | **-0.25** | **0.41** | **0.70** | **0.36** | **0.36** |
|  | area88 |  |  |  |  |  |  |  |  |  |  |  |  |  |  |  |  |  |  | 1.00 | **0.30** | **-0.31** | **-0.27** | **-0.41** | **-0.28** | 0.16 | **0.20** | **0.44** | 0.14 |
|  | area00 |  |  |  |  |  |  |  |  |  |  |  |  |  |  |  |  |  |  |  | 1.00 | **-0.22** | -0.13 | **-0.21** | **-0.19** | **0.19** | **0.35** | **0.35** | **0.67** |
|  | isol38 |  |  |  |  |  |  |  |  |  |  |  |  |  |  |  |  |  |  |  |  | 1.00 | **0.44** | **0.24** | **0.34** | **-0.39** | **-0.51** | **-0.29** | -0.11 |
|  | isol73 |  |  |  |  |  |  |  |  |  |  |  |  |  |  |  |  |  |  |  |  |  | 1.00 | **0.54** | **0.60** | -0.04 | -0.13 | -0.18 | 0.00 |
|  | isol88 |  |  |  |  |  |  |  |  |  |  |  |  |  |  |  |  |  |  |  |  |  |  | 1.00 | **0.76** | 0.10 | -0.11 | **-0.25** | 0.06 |
|  | isol00 |  |  |  |  |  |  |  |  |  |  |  |  |  |  |  |  |  |  |  |  |  |  |  | 1.00 | 0.04 | -0.07 | -0.10 | 0.10 |
|  | cont38 |  |  |  |  |  |  |  |  |  |  |  |  |  |  |  |  |  |  |  |  |  |  |  |  | 1.00 | **0.64** | 0.09 | **0.32** |
|  | cont73 |  |  |  |  |  |  |  |  |  |  |  |  |  |  |  |  |  |  |  |  |  |  |  |  |  | 1.00 | **0.33** | **0.39** |
|  | cont88 |  |  |  |  |  |  |  |  |  |  |  |  |  |  |  |  |  |  |  |  |  |  |  |  |  |  | 1.00 | **0.30** |
|  | cont00 |  |  |  |  |  |  |  |  |  |  |  |  |  |  |  |  |  |  |  |  |  |  |  |  |  |  |  | 1.00 |
